# Serum uromodulin and progression of kidney disease in patients with chronic kidney disease

**DOI:** 10.1186/s12967-018-1693-2

**Published:** 2018-11-19

**Authors:** Li Lv, Jinwei Wang, Bixia Gao, Liang Wu, Fang Wang, Zhao Cui, Kevin He, Luxia Zhang, Min Chen, Ming-Hui Zhao

**Affiliations:** 1 Renal Division, Department of Medicine, Peking University First Hospital; Institute of Nephrology, Peking University; Key Laboratory of Renal Disease, Ministry of Health of China; Key Laboratory of Chronic Kidney Disease Prevention and Treatment (Peking University), Ministry of Education, Beijing, 100034 China; 2 The First Affiliated Hospital, Baotou Medical College, Baotou, 014010 China; 3 Department of Biostatistics, School of Public Health, University of Michigan, Ann Arbor, MI USA; 4 Center for Data Science in Health and Medicine, Peking University, Beijing, China; 5 Peking-Tsinghua Center for Life Sciences, Beijing, China

**Keywords:** Uromodulin, Chronic kidney disease, Outcomes

## Abstract

**Background:**

Uromodulin is specifically synthesized and secreted by kidney tubular epithelial cells. Studies on the association of serum uromodulin and outcomes of chronic kidney disease (CKD) are lacking. This study aimed to evaluate whether serum uromodulin was associated with outcomes of patients with CKD.

**Methods:**

We measured serum uromodulin concentrations by ELISA in 2652 CKD patients from the Chinese Cohort Study of Chronic Kidney Disease (C-STRIDE) and investigated the association of serum uromodulin with outcomes of CKD patients, including end-stage kidney disease (ESKD) receiving kidney replacement therapy, cardiovascular events and mortality by Cox proportional hazards regression model.

**Results:**

A total of 2652 CKD patients were enrolled in this study, with an age of 48.7 ± 13.8 years and the baseline eGFR of 49.6 ± 29.4 mL/min/1.73 m^2^, of whom 58.4% were male. The median level of urinary albumin/creatinine ratio and serum uromodulin was 473.7 mg/g (IQR 134.1–1046.6 mg/g) and 77.2 ng/mL (IQR 48.3–125.9 ng/mL), respectively. Altogether, 404 ESKD, 189 cardiovascular events, and 69 deaths occurred during the median follow-up of 53.6 (IQR 44.0–64.0) months. Lower levels of serum uromodulin were independently associated with higher risk of incident ESKD after adjusting for traditional cardiovascular risk factors, with the hazard ratios (HRs) of 3.23 (95% confidence intervals [CIs] 2.15–4.85) for the middle tertile and 7.47 (95% CI 5.06–11.03) for the bottom tertile, compared with top tertile and 0.31 (95% CI 0.25–0.38) per every standard deviation increase. After further adjustment for the baseline eGFR, the association was greatly attenuated, but still significant, with HRs of 1.92 (95% CI 1.26–2.90) for the bottom tertile compared with top tertile and 0.69 (95% CI 0.55–0.86) per every standard deviation increase.

**Conclusions:**

Serum uromodulin is independently associated with an increased risk of incident ESKD in CKD patients.

**Electronic supplementary material:**

The online version of this article (10.1186/s12967-018-1693-2) contains supplementary material, which is available to authorized users.

## Background

Chronic kidney disease (CKD), with a high prevalence of over 10%, is one of the leading global public health problems [1]. In addition to progression to end-stage kidney disease (ESKD), patients with CKD are at high risk for cardiovascular disease and mortality [2]. It is thus particularly important to distinguish high-risk CKD patients. To date, biomarkers for evaluating the risk of CKD progression are restricted to the estimated glomerular filtration rate (eGFR) and proteinuria; these cannot fully reflect the tubulointerstitial function or the heterogeneity of CKD progression. Based on some previous studies, tubulointerstitial lesions might be more important for predicting kidney disease progression than glomerular and vascular lesions in patients with various kidney diseases [3–6]. The status of tubulointerstitium might better reflect total nephron mass and therefore could be helpful for identifying high-risk CKD patients more precisely. However, such kinds of markers are not well-established.

Uromodulin is the most abundant protein in urine [7–9] and is specifically synthesized and secreted by kidney tubular epithelial cells. Rare mutations in the *UMOD* gene have been described as a cause of hereditary autosomal-dominant tubulointerstitial diseases [10, 11]. Well-known genome-wide association studies (GWAS) have successfully identified common variants in the *UMOD* gene as risk factors for CKD and hypertension in the general population [12–14]. Recently, some prospective studies showed that serum uromodulin levels were independently associated with the risk of cardiovascular event and mortality as well as decline in kidney function among patients referred to angiography [15–17]. It is not yet known whether serum uromodulin was associated with outcomes of CKD patients. Hence, in the current study, we measured the serum uromodulin levels and investigated the association of serum uromodulin with kidney function and outcomes of CKD in a large, multicenter prospective cohort study of CKD patients, the Chinese Cohort Study of Chronic Kidney Disease (C-STRIDE).

## Methods

### Participants

The C-STRIDE is a multicenter prospective cohort of CKD patients, containing 39 clinical centers in different geographic regions of China. The criteria for the enrollment of participants are listed in Additional file 1. CKD participants have been enrolled from November 2011, a total of 3499 patients have completed screening until 30 June 2016, of which 686 patients were excluded due to missing values of serum creatinine and/or loss of follow-up data, altogether 2813 patients have the completed baseline and follow-up data. Due to the availability of the biosamples for measuring serum uromodulin, therefore, 2652 patients were included in the present study. For the etiologic diagnosis, there were 1707 patients with glomerular diseases, 547 patients with tubulointerstitial diseases and 398 patients with other or unknown causes. The design of C-STRIDE has been described elsewhere in detail [18].

### Study design

We measured serum uromodulin levels of 2652 CKD patients at baseline and described the distribution of baseline data of these patients according to serum uromodulin levels. Then, we further investigated the associations of serum uromodulin with pre-specified end-points of CKD patients, including ESKD, cardiovascular events and all-cause mortality. The baseline data included detailed demographics, underlying disease, behavioral habits, medical and medication history, anthropometric measures (height, weight, resting blood pressure), chemistry indexes (triglyceride, total cholesterol, low-density lipoprotein cholesterol, high-density lipoprotein cholesterol, fasting blood glucose, prealbumin, serum creatinine, high-sensitivity C-reactive protein), and urinary albumin/creatinine ratio. The end-point events of CKD patients were collected before 30 June 2017. All these patients gave written informed consent before data collection. The study was approved by the Ethics Committee of Peking University First Hospital and was in adherence with the Declaration of Helsinki.

### Detection of serum uromodulin by enzyme-linked immunosorbent assay (ELISA)

Fasting venous blood samples were obtained at the study visit. All participants’ blood samples were transported by cold chain to the Central Laboratory of Peking University First Hospital and stored at − 80 °C until use. Serum uromodulin was measured in batches from stored material in the Central Laboratory of Peking University First Hospital from May 2016 to August 2016. We measured serum uromodulin by a commercially available ELISA kit (Euroimmun AG, Lübeck, Germany) according to the manufacturer’s instructions. The process of the assay was described in Additional file 1.

### Measurement of covariates

The covariates in the multivariable models were either potential confounders, or the independent predictors of the adverse outcomes. All blood and urine samples were analyzed in the Central Laboratory of Peking University First Hospital to avoid the variation of testing values between laboratories. Serum total, low-density lipoprotein, and high-density lipoprotein cholesterol, triglycerides were measured with commercially available reagents. Urinary albumin and creatinine were measured from a fresh morning spot urine sample or morning urine sample stored at 4 °C for less than 1 week. Albuminuria was measured with immunoturbidimetric tests. Urinary creatinine was measured with the ammonia iminohydrolase method. The urinary albumin to creatinine ratio (mg/g creatinine) was calculated. Serum creatinine was measured by the same methods as urinary creatinine. The eGFR was evaluated by the equation developed by adaptation of the Modification of Diet in Renal Disease (MDRD) equation on the basis of data from the Chinese CKD participants: eGFR = 175 × (serum creatinine [in μmol/L]/88.4)^−1.234^ × age^−0.179^ × (if female × 0.79) [19]. All eGFR values of more than 120 mL/min/1.73 m^2^ were set at 120 mL/min/1.73 m^2^.

Body mass index was calculated by using the following formula: weight (kg)/height^2^ (m^2^). Blood pressure was measured three times at 5-min intervals by a sphygmomanometer. The mean value of the three readings was calculated. The use of anti-hypertensive medications in the past 2 weeks before baseline examination was recorded. Diabetes was defined as the fasting plasma glucose of 7.0 mmol/L or more, or the use of hypoglycemic agents or a self-reported history of diabetes.

### Study outcomes

The pre-specified end-point events of the CKD patients included ESKD, cardiovascular event, and all-cause mortality. ESKD is defined as the initiation of chronic dialysis or renal kidney transplantation or irreversible development of eGFR < 15 mL/min/1.73 m^2^. All the ESKD events included in the current study have initiated hemodialysis, or peritoneal dialysis, or kidney transplantation. Cardiovascular events included acute myocardial infarction, unstable angina, hospitalization for congestive heart failure, cerebrovascular events (intraparenchymal hemorrhage, subarachnoid hemorrhage, cerebral infarction, etc.), and peripheral vascular diseases. Echocardiogram and electrocardiogram examination were requested in the validation of cardiovascular events; however, cardio-angiography was not routinely performed. Only one event per patient was included in the current analysis. Finally, we separately evaluated the associations of baseline serum uromodulin with different clinical outcomes of CKD patients, including ESKD, cardiovascular events and all-cause mortality.

The end-point events were collected at 3- to 6-month intervals until 30 June 2017 in the current analysis. The director at each clinical center asked for end-point events according to the new-onset events registration form either by phone calls or routine clinical visits. Once the end-point events occurred, the director at the clinical center filled out the new-onset events registration form and submitted the related clinical data to the Renal Institute of Peking University via email within 1 month. The suspected clinical outcomes were then adjudicated by an independent committee consisting of specialist physicians.

### Statistical analysis

All CKD patients were stratified according to tertiles of baseline serum uromodulin levels. Continuous variables are presented as the means and standard deviations, except for highly skewed variables that are shown as median and interquartile ranges (IQR), and categorical variables are presented as proportions. One-way ANOVA was used to compare continuous variables, and Chi squared tests were used to compare categorical variables.

The incidence rates of end-point events (including ESKD, cardiovascular event and all-cause mortality) were calculated as number of events per 100 person-years. We depicted cumulative hazard function for the three events separately according to uromodulin levels by using a Kaplan–Meier curve and compared the event rates by using log-rank test.

To investigate the association between serum uromodulin and outcomes, Cox proportional hazards regression models were used to estimate hazards ratios (HRs) and 95% confidence intervals (CIs). We found that the risk of ESKD was linearly increased through the decline of uromodulin as shown in the linear spline analysis (Additional file 1: Figure S1), so we treated uromodulin either as a categorical variable (using the highest tertile as the reference) or a continuous variable (per standard deviation change) to represent the exposure variable. Multivariable models were constructed to adjust for potential confounding variables of the adverse outcomes, including age (continuous), gender (male vs. female), body-mass index (continuous), current smoker (yes vs. no), previous history of cardiovascular disease (yes vs. no), systolic blood pressure (continuous), using anti-hypertensive medications in the past 2 weeks (yes vs. no), diabetes (yes vs. no), prealbumin (continuous), logarithm transformed low-density lipoprotein cholesterol, high-density lipoprotein cholesterol [20], triglyceride, high-sensitivity C-reactive protein, urinary albumin/creatinine ratio (all in continuous), and eGFR (continuous). The missing values were filled before they were entered in the regression model. The proportional hazards assumption was assessed via Kaplan–Meier curves using log–log plots. We fitted logistic regression model by using the same covariates in the Cox regression model and calculated area under receiver operating characteristic curve (AUC). We compared AUCs inclusion or exclusion of serum uromodulin in the model with traditional cardiovascular risk factors, eGFR and ACR, in order to evaluate the change in discriminating ability for ESKD after inclusion of serum uromodulin. Statistical analyses were performed using the SAS software (version 9.4, SAS institute, CA, USA). *P* < 0.05 (two-sided) was considered statistically significant.

## Results

### Baseline characteristics by serum uromodulin levels

Among the 2652 CKD patients included in our study, 1548 (58.4%) were male, and 1104 (41.6%) were female, with an age of 48.7 ± 13.8 years. The baseline eGFR was 49.6 ± 29.4 mL/min/1.73 m^2^. A total of 788 (29.7%) participants had an eGFR greater than 60 mL/min/1.73 m^2^ at baseline. Altogether, 1053 (39.7%) and 811 (30.6%) patients were in CKD stages 3 and 4, respectively.

The median level of serum uromodulin was 77.2 ng/mL (IQR 48.3–125.9 ng/mL) in 2652 CKD patients. The levels in the three etiologic types of CKD were 100.4 ± 65.6 ng/mL in glomerular diseases, 69.0 ± 40.6 ng/mL in tubulointerstitial diseases and 73.4 ± 51.4 ng/mL in other or unknown causes, respectively (*P *< 0.001). The baseline characteristics of CKD patients according to tertiles of the serum uromodulin levels are presented in Table 1. Compared with patients with higher serum uromodulin levels, those with lower uromodulin levels were older, had a higher proportion of previous history of smoking, diabetes, and cardiovascular disease, higher proportion of current use of antihypertensive medications, and had higher levels of blood pressure, triglyceride, high-sensitivity C-reactive protein and urinary albumin/creatinine ratio but lower levels of total cholesterol and eGFR. The patients with missing value in the clinical characteristics tended to have a lower uromodulin level in our study. For example, the uromodulin levels were 79.20 ng/mL and 92.19 ng/mL, respectively, among those with and without missing value of systolic blood pressure (*P *< 0.001). In addition, we found a positive correlation between serum uromodulin and eGFR in multivariable linear correlation analysis (r = 0.68, *P *< 0.001).

**Table 1:** Baseline characteristics of the patients by tertiles of serum uromodulin

Characteristics	Total (N = 2652)	Serum uromodulin tertiles (ng/mL)		
		≤ 52.7 (N = 885)	> 52.7–100.8 (N = 881)	> 100.8 (N = 886)
Age (years)	48.7 ± 13.8	50.2 ± 13.6	50.0 ± 13.2	45.9 ± 14.1
Male (n%)	1548 (58.4%)	513 (58.0%)	534 (60.6%)	501 (56.6%)
Body mass index (kg/m^2^)	24.4 ± 3.6	24.2 ± 3.7	24.7 ± 3.7	24.4 ± 3.5
Systolic blood pressure (mmHg)	130.1 ± 19.3	135.7 ± 20.5	130.4 ± 18.4	124.7 ± 17.7
Diastolic blood pressure (mmHg)	81.3 ± 11.7	83.6 ± 12.4	81.6 ± 11.7	78.8 ± 10.4
Smoking status (n%)	907 (38.1%)	325 (42.4%)	300 (38.2%)	282 (34.0%)
Diabetes (n%)	516 (19.6%)	178 (20.3%)	202 (23.1%)	136 (15.4%)
Using anti-hypertensive medications in the past 2 weeks (n%)	1488 (73.2%)	540 (82.3%)	524 (76.7%)	424 (61.1%)
Cardiovascular disease (n%)	293 (12.2%)	121 (15.6%)	114 (14.2%)	58 (7.0%)
Triglyceride (mmol/L)	1.8 (1.3, 2.6)	1.8 (1.3, 2.6)	1.8 (1.3, 2.7)	1.7 (1.2, 2.4)
Total cholesterol (mmol/L)	4.8 (4.0, 5.8)	4.5 (3.7, 5.4)	4.9 (4.1, 5.9)	5.0 (4.1, 6.2)
High-density lipoprotein cholesterol (mmol/L)	1.1 (0.9, 1.3)	1.0 (0.9, 1.2)	1.1 (0.9, 1.3)	1.2 (1.0, 1.4)
Low-density lipoprotein cholesterol (mmol/L)	2.6 (2.1, 3.3)	2.4 (2.0, 3.0)	2.6 (2.1, 3.3)	2.8 (2.2, 3.5)
Fasting blood glucose (mmol/L)	4.9 (4.4, 5.6)	4.9 (4.4, 5.6)	5.0 (4.4, 5.7)	4.9 (4.4, 5.5)
Prealbumin (g/L)	326.5 ± 83.1	348.7 ± 87.6	327.6 ± 79.3	303.2 ± 75.6
High sensitive–reactive protein (mg/L)	1.3 (0.5, 3.0)	1.5 (0.6, 3.6)	1.4 (0.6, 3.1)	1.0 (0.4, 2.5)
Urinary albumin/creatinine ratio (mg/g)	473.7 (134.1, 1046.6)	661.7 (233.9, 1352.8)	439.9 (113.2, 969.9)	374.3 (100.0, 836.6)
Creatinine (μmol/L)	173.8 ± 121.8	247.4 ± 138.6	165.7 ± 114.5	108.4 ± 49.1
Estimated glomerular filtration rate (mL/min/1.73 m^2^)	49.6 ± 29.4	28.5 ± 15.0	46.3 ± 23.0	74.0 ± 28.0
Estimated glomerular filtration rate group (mL/min/1.73 m^2^)				
≥ 90	337 (12.7%)	5 (0.6%)	58 (6.6%)	274 (30.9%)
60–89	451 (17.0%)	31 (3.5%)	111 (12.6%)	309 (34.9%)
45–59	419 (15.8%)	73 (8.3%)	211 (24.0%)	135 (15.2%)
30–44	634 (23.9%)	211 (23.8%)	297 (33.7%)	126 (14.2%)
15–29	811 (30.6%)	565 (63.8%)	204 (23.2%)	42 (4.7%)

Missing counts: body mass index: 396, systolic blood pressure: 492, diastolic blood pressure: 492, smoking status: 271, diabetes: 16, using anti-hypertensive medications in the past 2 weeks: 619, cardiovascular disease: 240, triglyceride: 64, total cholesterol: 68, high-density lipoprotein cholesterol: 80, low-density lipoprotein cholesterol: 81, fasting blood glucose: 96, prealbumin: 151, high sensitive-reactive protein: 606, urinary albumin/creatinine ratio: 299

### The incidence rates of the end-point events according to levels of serum uromodulin

**Fig. 1:**
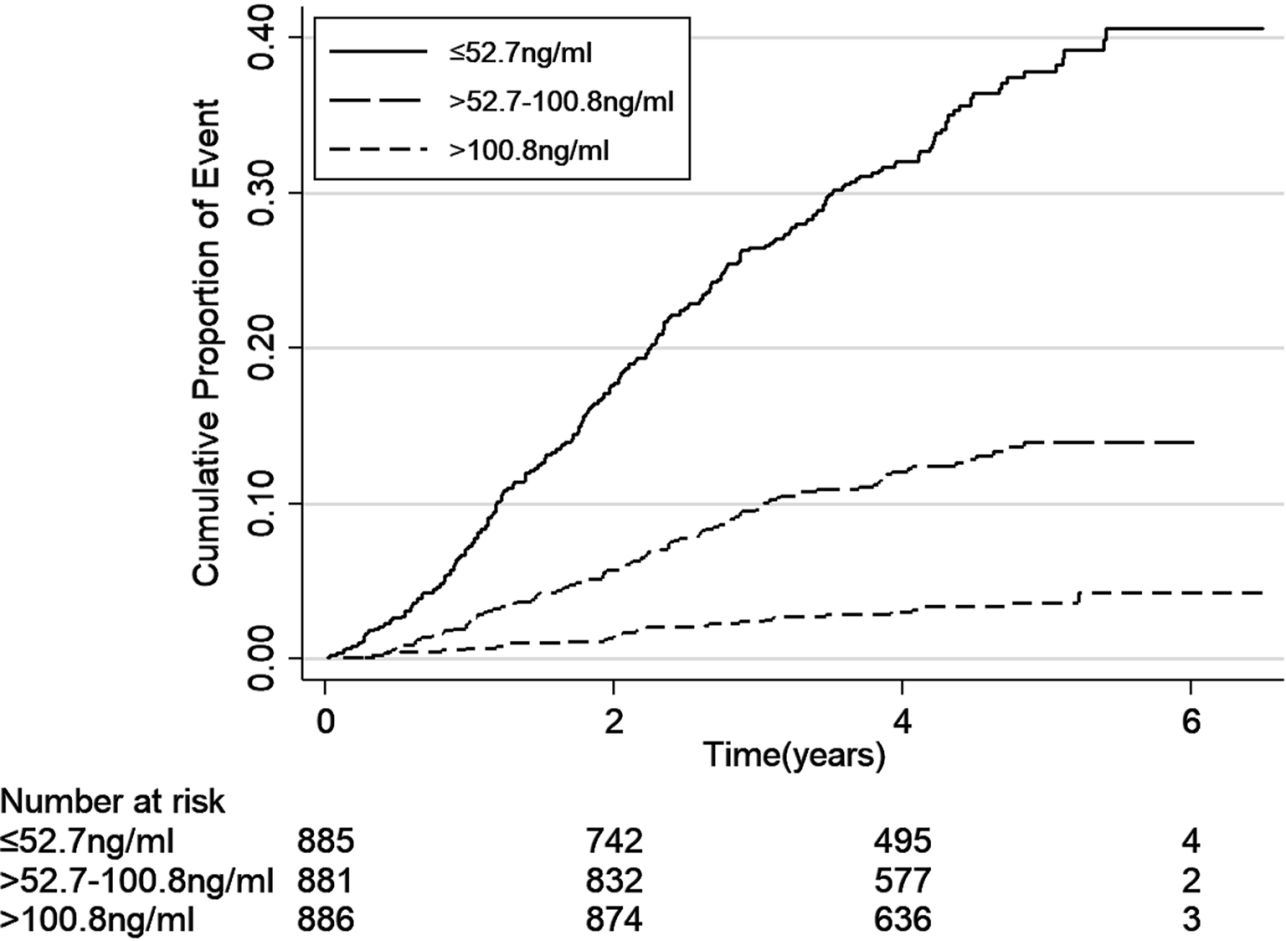
Kaplan–Meier curve for ESKD events according to tertiles of serum uromodulin

**Fig. 2:**
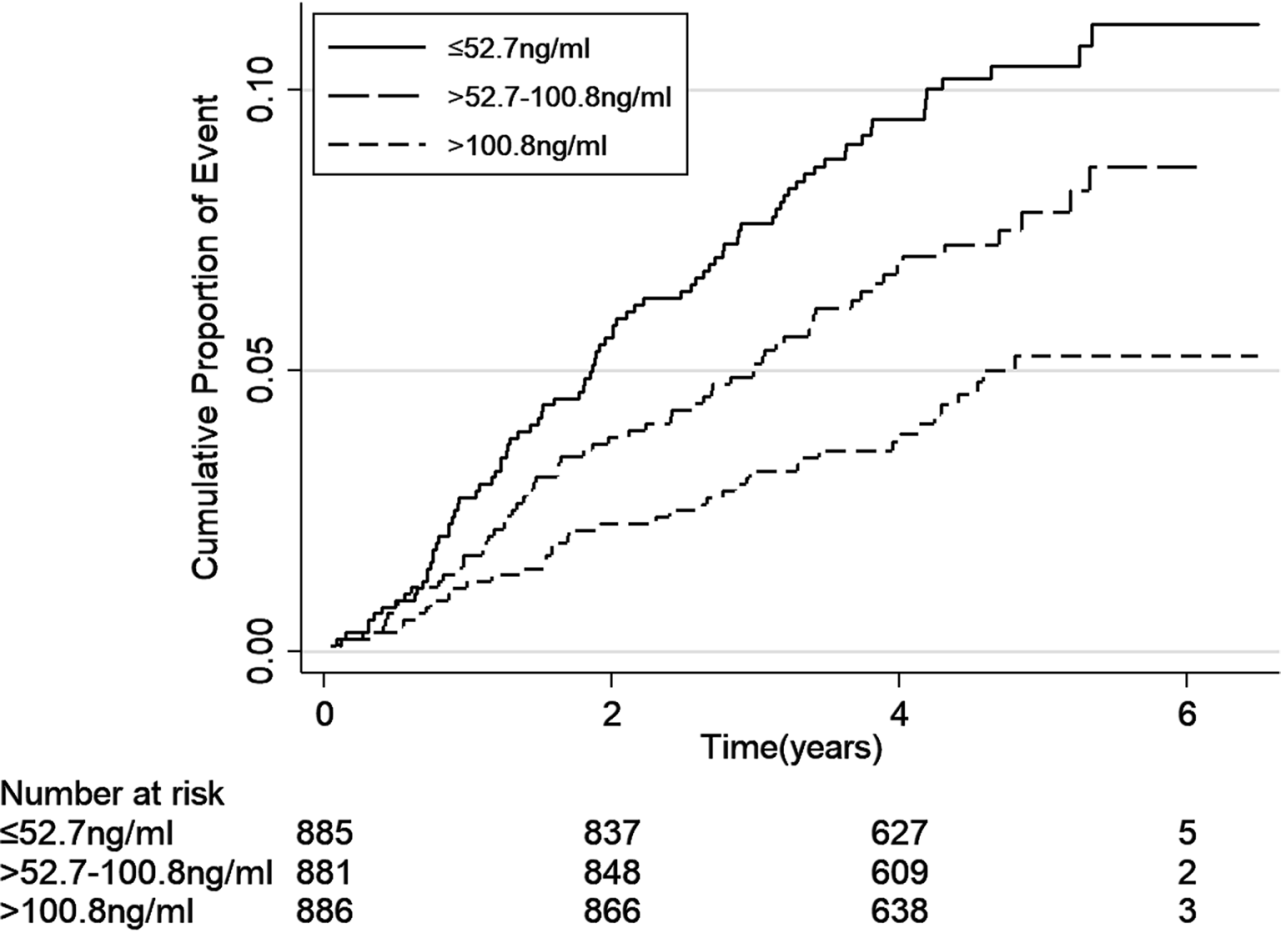
Kaplan–Meier curve for cardiovascular events according to tertiles of serum uromodulin

**Fig. 3:**
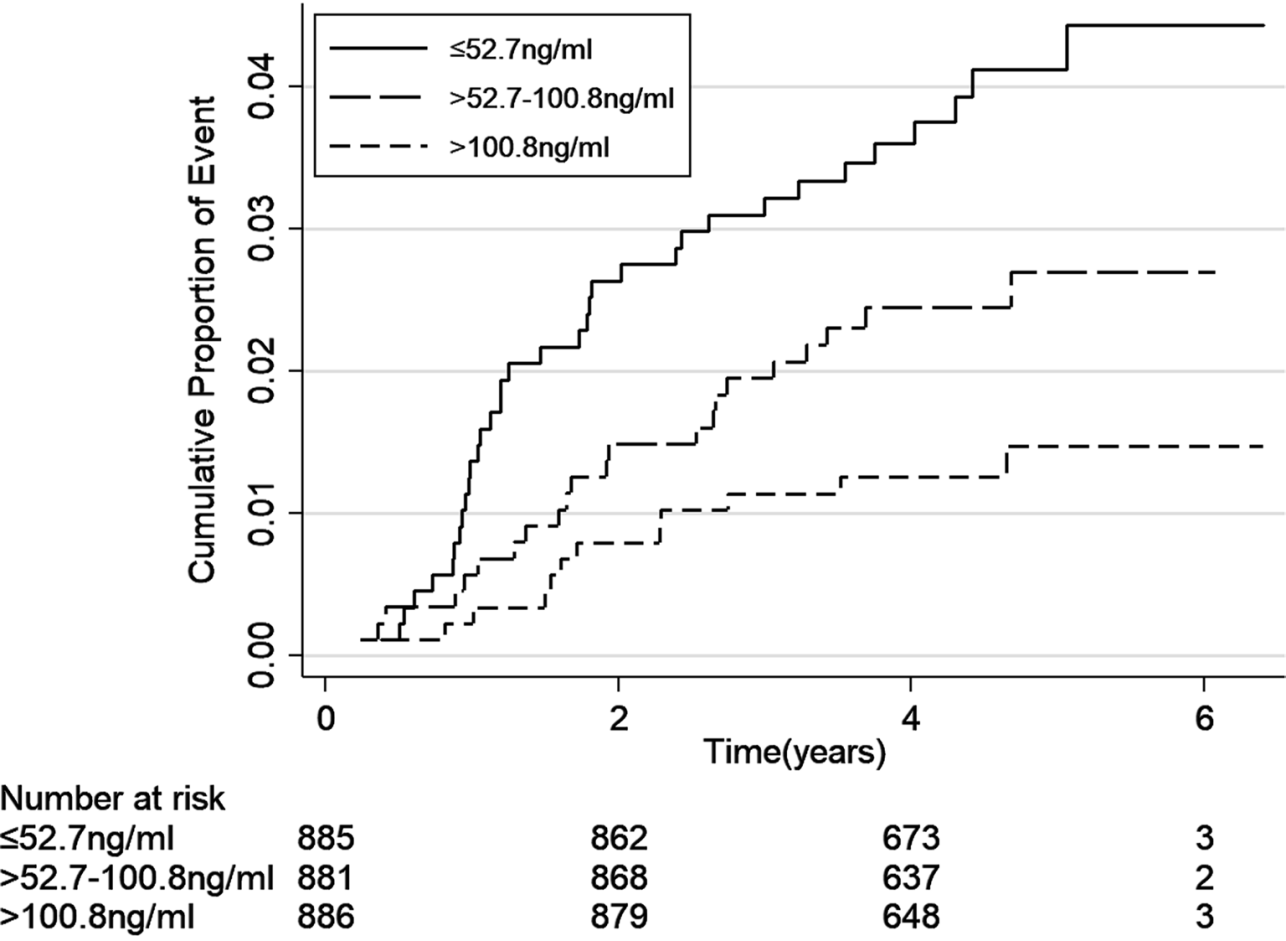
Kaplan–Meier curve for all-cause mortality according to tertiles of serum uromodulin

The incidence rates of end-point events according to levels of serum uromodulin are shown in Table 2. During the median follow-up of 53.6 (IQR 44.0–64.0) months, there were 404 ESKD, 189 cardiovascular events and 69 deaths occurred. ESKD, cardiovascular events and death rates were 3.60, 1.60 and 0.57 per 100 person-years, respectively. Higher incidence rates of all the three end-point events were seen with the decreased levels of uromodulin (Figs. 1, 2, 3, all P-values for log-rank test < 0.05).

**Table 2:** Association between the uromodulin levels and the end-point events rates

Serum uromodulin tertiles (ng/mL)	Number of events	Events per 100 person-years	*P* for log-rank
ESRD events			< 0.001
≤ 52.7 (N = 885)	267 (30.17%)	7.86	
> 52.7–100.8 (N = 881)	106 (12.03%)	2.80	
> 100.8 (N = 886)	31 (3.50%)	0.77	
Total	404 (15.23%)	3.60	
Cardiovascular events			< 0.001
≤ 52.7 (N = 885)	86 (9.72%)	2.20	
> 52.7–100.8 (N = 881)	63 (7.15%)	1.62	
> 100.8 (N = 886)	40 (4.51%)	0.99	
Total	189 (7.13%)	1.60	
All-cause mortality			0.003
≤ 52.7 (N = 885)	35 (3.95%)	0.87	
> 52.7–100.8 (N = 881)	22 (2.50%)	0.55	
> 100.8 (N = 886)	12 (1.35%)	0.29	
Total	69 (2.60%)	0.57

### Associations of serum uromodulin with ESKD, cardiovascular events and all-cause mortality

The association of serum uromodulin with outcomes is shown in Table 3. After adjusting for demographic and traditional cardiovascular risk factors, as well as the baseline eGFR levels, baseline serum uromodulin levels were independently associated with the risk of incident ESKD, with an HR of 1.92 (95% CI 1.26–2.90) in the bottom tertile compared with the top tertile. Every standard deviation increase of uromodulin was associated with a decreased risk of ESKD, with an HR of 0.69 (95% CI 0.55–0.86). However, we did not detect significant associations between serum uromodulin and the risk of cardiovascular events as well as all-cause mortality in multivariable adjusted model. Similar results were found among subgroup of patients with glomerular diseases and tubulointerstitial diseases (Additional file 1: Tables S1, S2).

**Table 3:** Association of serum uromodulin with ESKD, cardiovascular events and all-cause mortality

Serum uromodulin tertiles (ng/mL)	Model 1	Model 2	Model 3
	HR (95% CI)	HR (95% CI)	HR (95% CI)
ESKD events			
> 100.8	1.00 (Ref)	1.00 (Ref)	1.00 (Ref)
> 52.7–100.8	3.70 (2.48, 5.54)	3.23 (2.15, 4.85)	1.36 (0.90, 2.06)
≤ 52.7	10.46 (7.20, 15.21)	7.47 (5.06, 11.03)	1.92 (1.26, 2.90)
Per SD increase	0.26 (0.21, 0.32)	0.31 (0.25, 0.38)	0.69 (0.55, 0.86)
Cardiovascular events			
> 100.8	1.00 (Ref)	1.00 (Ref)	1.00 (Ref)
> 52.7–100.8	1.37 (0.92, 2.04)	1.04 (0.70, 1.56)	0.80 (0.52, 1.22)
≤ 52.7	1.85 (1.27, 2.70)	1.24 (0.83, 1.86)	0.81 (0.51, 1.28)
Per SD increase	0.71 (0.59, 0.87)	0.88 (0.72, 1.08)	1.14 (0.89, 1.45)
All-cause mortality			
> 100.8	1.00 (Ref)	1.00 (Ref)	1.00 (Ref)
> 52.7–100.8	1.63 (0.80, 3.29)	1.35 (0.65, 2.77)	1.04 (0.48, 2.23)
≤ 52.7	2.59 (1.34, 5.00)	1.94 (0.96, 3.94)	1.30 (0.58, 2.93)
Per SD increase	0.63 (0.45, 0.89)	0.73 (0.51, 1.04)	0.92 (0.60, 1.41)

Model 1: Adjusted for age, gender

Model 2: Model 1+ current smoker, body-mass index, diabetes, systolic blood pressure, using anti-hypertensive medications in the past 2 weeks, cardiovascular diseases history, logarithm transformed triglyceride, logarithm transformed low-density lipoprotein cholesterol, prealbumin, logarithm transformed high-density lipoprotein cholesterol, logarithm transformed sensitive-reactive protein and logarithm transformed urinary albumin/creatinine ratio

Model 3: Model 2+ estimated glomerular filtration rate

*ESKD* end stage kidney disease, *SD* standard deviation

In the fully adjusted logistic regression model for ESKD, the AUCs with inclusion or exclusion of serum uromodulin level were 0.8623 (95% CI 0.8438–0.8808) and 0.8601 (95% CI 0.8413–0.8789), respectively. The difference of the AUCs was 0.0023 (95% CI − 0.0007 to 0.0052) (P-value for the difference = 0.1).

## Discussion

In the current study, we described the association of serum uromodulin and ESKD, cardiovascular events, all-cause mortality in the context of CKD. We found that the baseline lower levels of serum uromodulin were associated with an increased risk of incident ESKD independent of the traditional risk factors for progression of CKD.

Uromodulin is a glycosyl phosphatidylinositol (GPI) linked glycoprotein exclusively synthesized in renal tubular epithelial cells [21]. Most of the uromodulin protein cleaved by proteolysis is released into the urine [22, 23]; a smaller but significant basolateral release of uromodulin is secreted to the tubulointerstitium [3, 24–26] and is detected in the blood. Recently, two SNPs within the promoter region of the *UMOD* gene were found to be associated with a decline in the occurrence of CKD and a lower urinary uromodulin level [12, 13]. Previous studies mainly focused on urinary uromodulin excretion [8, 25, 27], but serum uromodulin has not been investigated widely. Furthermore, serum uromodulin is a stable monomeric antigen and seem to be more reliably measured [28]. Thus, we evaluated whether the level of serum uromodulin was associated with outcomes of CKD in a large, multicenter prospective cohort study of CKD patients.

To the best of our knowledge, the current study is the first prospective one assessing the association between serum uromodulin and kidney disease progression in CKD population. Our results were, to some extent, in line with several recent studies [17, 29]. A recent cross-sectional study by Steubl et al. presented that plasma uromodulin could identify early stages of CKD [29]. Additionally, Leiherer et al. [17] reported that lower levels of serum uromodulin were independently associated with the decline of kidney function and the incidence of CKD in patients with established or suspected stable coronary artery disease. However, most of participants included in their study had normal and moderately reduced kidney function and failed to reach the endpoint of ESKD during follow-up. Thus, we extended the previous observation by a large, prospective Chinese CKD cohort with a broad range of eGFR.

Uromodulin is specifically secreted by renal tubular epithelial cells. Experimental data from Trudu [14] showed that over-expression of uromodulin leads to salt-sensitive hypertension, left ventricular hypertrophy and kidney damage. SNPs leading to overexpression of uromodulin in humans are strongly associated with a greater risk of CKD [13]. The above situation is based on individuals with physically functional kidney and variation of uromodulin exits due to the genetic background among individuals. However, in the setting of abnormal kidney function/kidney damage, the decline in absolute uromodulin excretion was caused by the reduction in functional nephron mass and/or reserve of the tubules [11, 30]. It is possible that a decline in urinary uromodulin excretion from apical secretion is associated with a decline in basolateral release, as evidenced by a decline in the level of serum uromodulin in CKD patients [31, 32]. Thus, lower levels of serum uromodulin indirectly reflected abnormalities in renal tubulointerstitial function, which is associated with a reduction in erythropoietin production, acid–base homeostasis disequilibrium and mineral metabolism disorder, which therefore links the rapid progress on kidney function.

Although the physiological role of circulating uromodulin remains largely unknown, the present data strengthens the notion that serum uromodulin represents a marker of kidney health independent of markers of the glomerular function and might help to distinguish high-risk CKD patients with rapid progress on kidney function. With regard to the ESKD events, our results only found a trend of improvement in the discriminating ability after inclusion of serum uromodulin in the model with traditional markers for kidney function and kidney damage. With regard to the cardiovascular complications, Leiherer et al. and Delgado et al. reported that serum uromodulin could predict the risk of cardiovascular events and all-cause mortality among people with coronary disease [15, 16]. However, we did not observe similar associations in our study. The different ethnic background and a lower cardiovascular risk profile (younger age, lower levels of blood pressure and lower prevalence of diabetes mellitus) might be the reasons for such inconsistency.

There are some limitations of the current study. First, the cohort has a relatively short duration of follow-up and a limited number of cardiovascular events and death, which limited our power to investigate the association between the levels of uromodulin and cardiovascular disease. Furthermore, some laboratory tests for cardiovascular disease, including NT-proBNP, hs-Troponin and Galectin-binding protein, were not included in our study. Second, although most well-established risk factors of CKD progression were included in the multivariable regression models, the possibility of residual confounding still exists. Finally, considering the baseline characteristics of our study, these results will be primarily applicable to patients seen by nephrologists rather than the large population with CKD in the general population who have lower levels of proteinuria and older age.

## Conclusions

This study represents the first prospective cohort study with a large sample size investigating the association between serum uromodulin and outcomes in CKD population. Serum uromodulin levels are independently associated with incident ESKD.

## Additional file

**Additional file 1.** Additional figure and tables.

## Abbreviations

CKD: chronic kidney disease
C-STRIDE: The Chinese Cohort Study of Chronic Kidney Disease
ESKD: end-stage kidney disease
HRs: hazard ratios
eGFR: estimated glomerular filtration rate
IQR: interquartile ranges
CIs: confidence intervals
GPL: glycosyl phosphatidylinositol
