# Supplementary material for: Serum uromodulin and progression of kidney disease in patients with chronic kidney disease
Source: J Transl Med. 2018 Nov 19;16:316. doi: 10.1186/s12967-018-1693-2 (PMC6245763; doi:10.1186/s12967-018-1693-2)
Supplement: Supplementary file 1 — Additional file 1. Additional figure and tables. [file 12967_2018_1693_MOESM1_ESM.doc]

**Additional material**

**Methods**

**The criteria for the enrollment of participants**

Participants met the following criteria to be eligible for enrollment: (1) aged between 18 and 74 years and (2) specified eGFR range according to different CKD etiologies. For patients with glomerulonephritis (GN), the eGFR should be ≥ 15ml·min-1·1.73 m^-2^. For patients with diabetic nephropathy (DN), the defining eligibility was 15ml·min-1·1.73m^-2^ ≤ eGFR < 60ml·min-1·1.73m^-2^ or eGFR ≥ 60ml·min-1·1.73 m^-2^ with “nephrotic range” proteinuria, which is defined as 24-hour urinary protein ≥ 3.5g or urinary albumin creatinine ratio (ACR) ≥ 2000mg/g or corresponding values of urine dipstick test or urinary protein creatinine ratio (PCR). For non-GN and non-DN patients, 15ml·min-1·1.73m^-2^ ≤ eGFR < 60ml·min-1·1.73m^-2^ is setting for enrollment.

**The process of the ELISA assay for testing serum uromodulin**

A 96-well plate was coated with uromodulin in advance and blocked to reduce non-specific binding. The serum samples were diluted 1:101 using dilution buffer, i.e., dilute a 10μL serum sample in 1.0mL sample buffer and mix well by vortexing. A total of 100μL of calibrators, controls, or diluted patient samples were pipetted into coated wells of the microtiter plate; subsequently, 100μL of biotinylated detection antibody were added. The microtiter plate was covered with foil and was incubated for 2 hours at room temperature at 450 rotations per minute (rpm) on an orbital shaker. Following three washes of the plate using 300μL washing buffer, wash buffer in each well was left for 30 to 60 seconds per washing cycle, and then, the wells were tapped gently on absorbent paper. A total of 100μL of streptavidin-peroxidase was pipetted into each well followed by incubation for 30 minutes at 450 rpm. Subsequently, the streptavidin-peroxidase was soaked, and the microtiter plate was washed three times. Consequently, 100μL of substrate solution was pipetted into each well. The microtiter plate was incubated in the dark for 15 minutes at room temperature. The reaction was terminated by adding 100μL of stop solution. This causes a color change from blue to yellow. Finally, the substrate solution was measured using a photometer at a wavelength of 450nm and a reference wave length of 620nm. For this assay, at a mean concentration of 29.7ng/mL, 102.0ng/mL and 214.4ng/mL, the intra-assay coefficient of variation was 3.2%, 2.2% and 1.8%, respectively. The lower detection limit of the uromodulin ELISA was 2.0ng/mL.


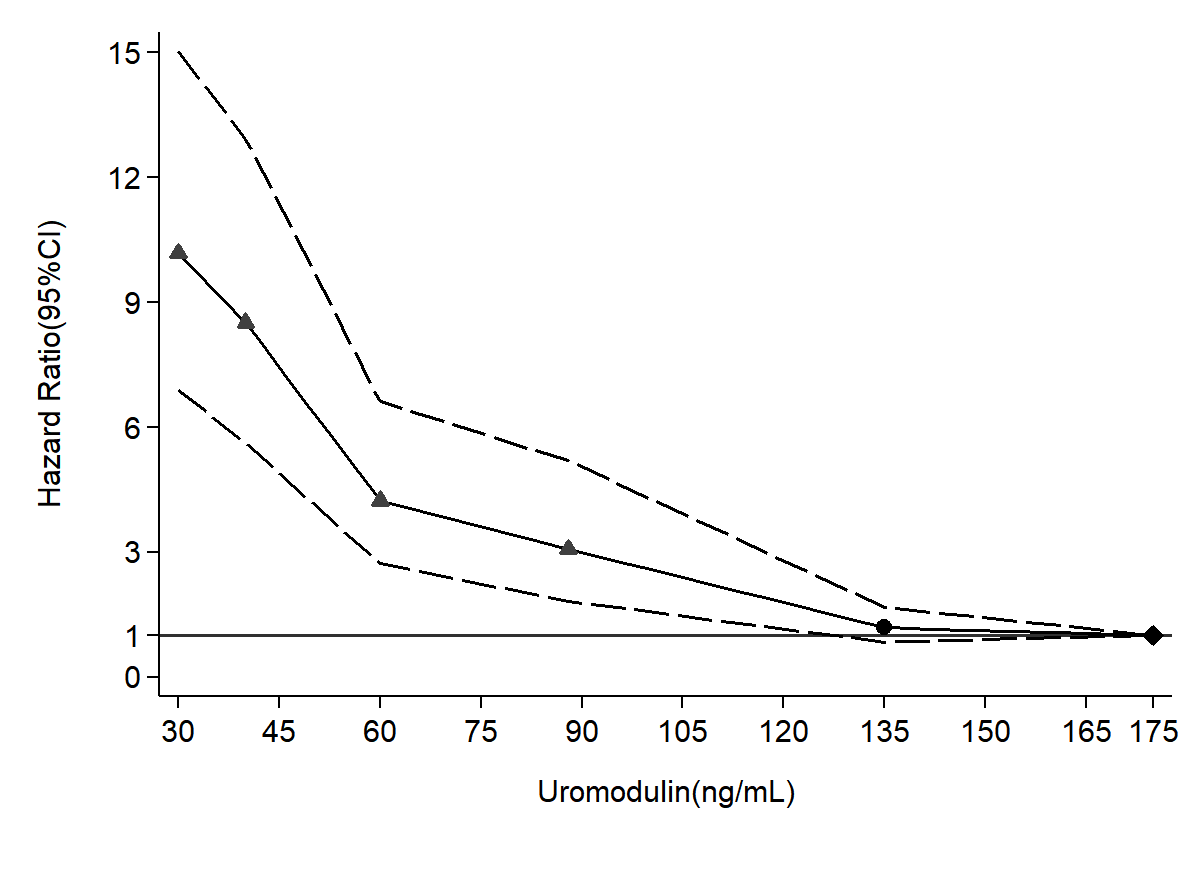


**Additional file 1: Figure S1.** Hazard ratios and 95% confidence intervals for end stage kidney disease according to spline uromodulin.

Uromodulin was treated as a linear spline variable with knots at 20%, 40%, 60% and 80% percentiles of the variable. The association of uromodulin with ESKD seems continuously increasing with the decline of uromodulin (with 175 ng/mL as reference). Hazard ratios and 95% confidence intervals (area between dash lines) in age and gender adjusted Cox hazards regression model. The reference (diamond) was uromodulin 175ng/mL. Triangles represent statistically significant and circles represent not significant.

| **Additional file 1: table S1.** Association of serum uromodulin with ESKD, cardiovascular events and all-cause mortality among glomerular disease (n=1707) | | | | |
| --- | --- | --- | --- | --- |
| Serum uromodulin | Model 1 | Model 2 | Model 3 | |
| tertiles (ng/mL) | HR (95% CI) | HR (95% CI) | HR (95% CI) | |
| ESKD events |  |  |  | |
| >115.7 | 1.00 (Ref) | 1.00 (Ref) | 1.00 (Ref) | |
| >59.7-115.7 | 3.34(2.02,5.52) | 2.90(1.74,4.82) | 1.28(0.76,2.15) | |
| ≤59.7 | 10.93(6.87,17.41) | 7.03(4.32,11.43) | 1.66(0.99,2.79) | |
| per SD increase | 0.24(0.18,0.30) | 0.31(0.24,0.40) | 0.75(0.58,0.95) | |
| Cardiovascular events |  |  |  | |
| >115.7 | 1.00 (Ref) | 1.00 (Ref) | 1.00 (Ref) | |
| >59.7-115.7 | 0.65(0.36,1.16) | 0.50(0.28,0.90) | 0.39(0.21,0.73) | |
| ≤59.7 | 1.89(1.19,3.00) | 1.13(0.68,1.87) | 0.75(0.41,1.36) | |
| per SD increase | 1.02(0.78,1.32) | 1.35(0.99,1.85) | 1.35(0.99,1.85) | |
| All-cause mortality |  |  |  | |
| >115.7 | 1.00 (Ref) | 1.00 (Ref) | 1.00 (Ref) | |
| >59.7-115.7 | 1.00(0.36,2.78) | 0.94(0.33,2.67) | 0.75(0.25,2.24) | |
| ≤59.7 | 3.45(1.49,7.96) | 2.97(1.18,7.47) | 2.01(0.68,5.94) | |
| per SD increase | 0.55(0.35,0.86) | 0.61(0.38,0.99) | 0.79(0.45,1.39) | |
| Model 1: Adjusted for age, gender; | | | | |
| Model 2: Model 1+current smoker, body-mass index, diabetes, systolic blood pressure, using anti-hypertensive medications in the past two weeks, cardiovascular diseases history, logarithm transformed triglyceride, logarithm transformed low-density lipoprotein cholesterol, prealbumin, logarithm transformed high-density lipoprotein cholesterol, logarithm transformed sensitive-reactive protein and logarithm transformed urinary albumin/creatinine ratio; | | | | |
| Model 3: Model 2+ estimated glomerular filtration rate. | | | | |
| [Abbreviations]ESKD: end stage kidney disease; SD: standard deviation. | | | |  |

| **Additional file 1: table S2.** Association of serum uromodulin with ESKD, cardiovascular events and all-cause mortality among tubulointerstitial diseases (n=547) | | | |
| --- | --- | --- | --- |
| Serum uromodulin | Model 1 | Model 2 | Model 3 |
| tertiles (ng/mL) | HR (95% CI) | HR (95% CI) | HR (95% CI) |
| ESKD events |  |  |  |
| >80.7 | 1.00 (Ref) | 1.00 (Ref) | 1.00 (Ref) |
| >46.8-80.7 | 2.69(1.19,6.07) | 2.26(0.97,5.23) | 1.41(0.59,3.34) |
| ≤46.8 | 8.96(4.27,18.80) | 5.95(2.75,12.87) | 2.46(1.08,5.57) |
| per SD increase | 0.32(0.22,0.45) | 0.40(0.27,0.58) | 0.64(0.43,0.94) |
| Cardiovascular events |  |  |  |
| >80.7 | 1.00 (Ref) | 1.00 (Ref) | 1.00 (Ref) |
| >46.8-80.7 | 2.35(1.06,5.20) | 2.13(0.94,4.83) | 2.04(0.89,4.71) |
| ≤46.8 | 2.24(1.00,5.04) | 1.70(0.72,4.02) | 1.51(0.57,4.00) |
| per SD increase | 0.70(0.49,1.01) | 0.78(0.54,1.14) | 0.80(0.52,1.24) |
| All-cause mortality |  |  |  |
| >80.7 | 1.00 (Ref) | 1.00 (Ref) | 1.00 (Ref) |
| >46.8-80.7 | 0.87(0.26,2.84) | 0.76(0.22,2.58) | 0.57(0.16,2.01) |
| ≤46.8 | 1.47(0.51,4.25) | 0.94(0.30,2.97) | 0.51(0.14,1.91) |
| per SD increase | 0.65(0.36,1.17) | 0.80(0.44,1.43) | 0.99(0.51,1.90) |
| Model 1: Adjusted for age, gender; | | | |
| Model 2: Model 1+current smoker, body-mass index, diabetes, systolic blood pressure, using anti-hypertensive medications in the past two weeks, cardiovascular diseases history, logarithm transformed triglyceride, logarithm transformed low-density lipoprotein cholesterol, prealbumin, logarithm transformed high-density lipoprotein cholesterol, logarithm transformed sensitive-reactive protein and logarithm transformed urinary albumin/creatinine ratio; | | | |
| Model 3: Model 2+ estimated glomerular filtration rate. | | | |
| [Abbreviations]ESKD: end stage kidney disease; SD: standard deviation. | | | |
